# Visual predictions, neural oscillations and naïve physics

**DOI:** 10.1038/s41598-021-95295-x

**Published:** 2021-08-09

**Authors:** Blake W. Saurels, Wiremu Hohaia, Kielan Yarrow, Alan Johnston, Derek H. Arnold

**Affiliations:** 1 School of Psychology, The University of Queensland, Brisbane, Australia; 2 Department of Psychology, City, University of London, London, UK; 3 School of Psychology, University of Nottingham, Nottingham, UK

**Keywords:** Visual system, Sensory processing

## Abstract

Prediction is a core function of the human visual system. Contemporary research suggests the brain builds predictive internal models of the world to facilitate interactions with our dynamic environment. Here, we wanted to examine the behavioural and neurological consequences of disrupting a core property of peoples’ internal models, using naturalistic stimuli. We had people view videos of basketball and asked them to track the moving ball and predict jump shot outcomes, all while we recorded eye movements and brain activity. To disrupt people’s predictive internal models, we inverted footage on half the trials, so dynamics were inconsistent with how movements should be shaped by gravity. When viewing upright videos people were better at predicting shot outcomes, at tracking the ball position, and they had enhanced alpha-band oscillatory activity in occipital brain regions. The advantage for predicting upright shot outcomes scaled with improvements in ball tracking and occipital alpha-band activity. Occipital alpha-band activity has been linked to selective attention and spatially-mapped inhibitions of visual brain activity. We propose that when people have a more accurate predictive model of the environment, they can more easily parse what is relevant, allowing them to better target irrelevant positions for suppression—resulting in both better predictive performance and in neural markers of inhibited information processing.

## Introduction

The capacity to form predictions about the world is seen as a core function of the visual brain^1–3^. These predictions allow us to catch flying balls and dodge fast moving projectiles. It has been suggested that the brain accomplishes these feats by forming predictive internal models of the environment^2,4^, which could sometimes be in persistent error^5^.

One way contemporary research has probed these ideas is by looking at eye movements^6^. Hayhoe et al.^6^ examined the role of prediction in gaze behaviour while playing squash. Their data suggested the brain can construct a dynamic predictive model for how a squash ball will move, which it uses to guide predictive eye movements. Similar effects have been demonstrated with people playing cricket^7,8^ and baseball^9^. In this study we aimed to build on these investigations, by incorporating a simultaneous measure of neural activity (electroencephalography; EEG) while people performed a predictive task concerning naturalistic inputs.

**Figure 1:**
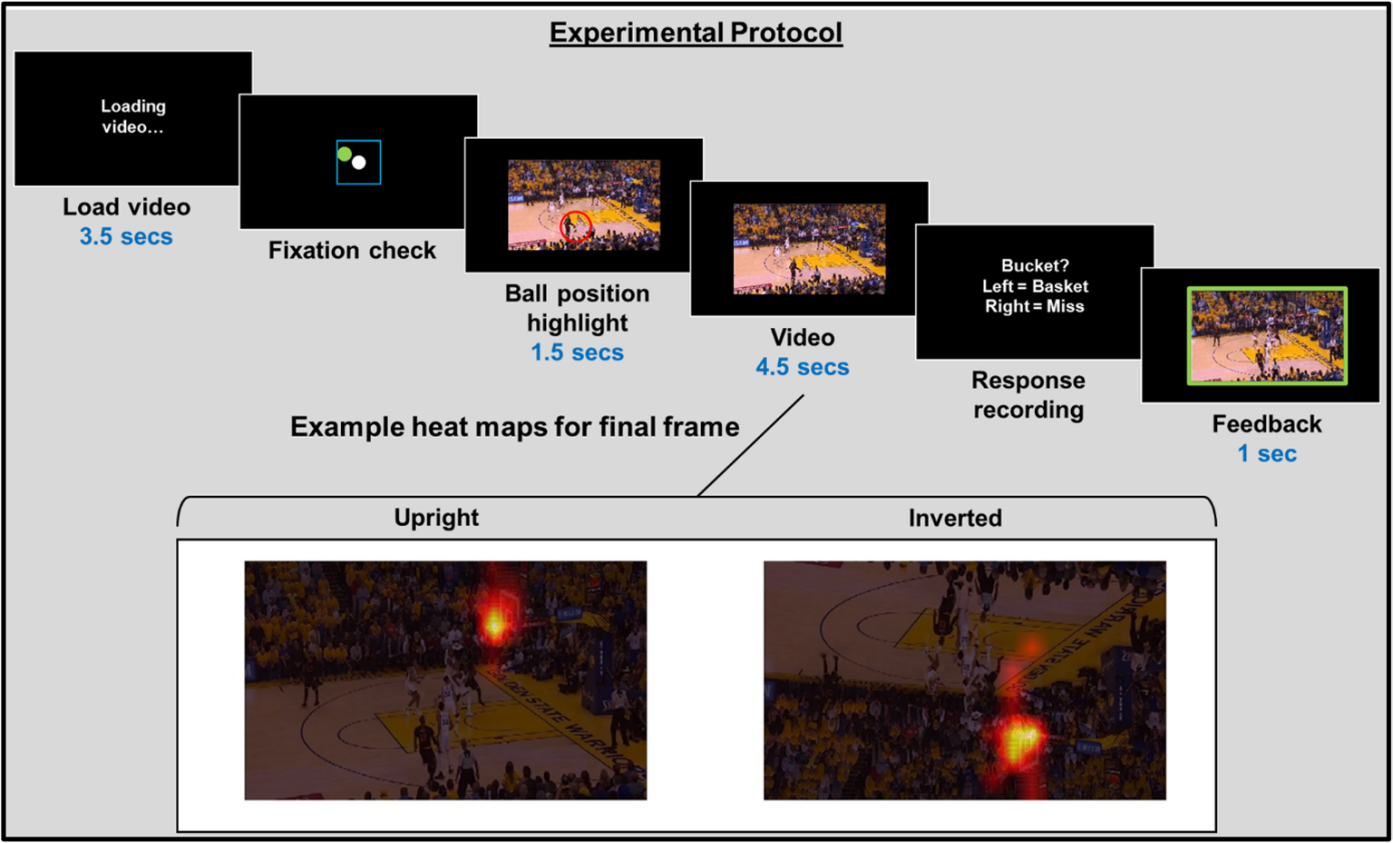
Graphic depicting experimental protocol, and example heat maps for final frame of a video. Heat map created using Matlab software.

We used videos of basketball, each culminating in a jump shot (see Fig. 1). We created a predictive task by cutting off footage just as the ball neared the basket, and had participants predict the shot outcome. Participants were asked to track the ball as it moved for a few seconds leading up to a jump shot while we recorded their’ eye movements and took electroencephalogram (EEG) recordings.

To manipulate peoples’ ability to predict shot outcomes, we presented videos upright—a situation that accords with peoples’ intuitive understanding of physics^10–12^, or upside-down, a situation that conflicts with expectations. We reasoned that this disruption of peoples’ understanding of how scene dynamics should be impacted by gravity should undermine the formation of accurate predictive models of the environment, and thereby negatively impact shot outcome predictions.

In terms of a plausible neural correlate of successful visual predictions, we were particularly interested in alpha-band (8–12 Hz) oscillatory activity in occipital brain regions. These have been implicated as a marker of selective attention^13–17^, potentially driven by feedback to deep layers of visual cortex^18^. The operations we envisage could be characterised as a dynamic spatially targeted inhibition of visual information processing^17,19,20^; although, see^21^.

We anticipated that if people have a more accurate predictive model of the dynamics of the environment, in our task they should be better able to locate and predict the trajectory of a moving ball—resulting in better ball tracking and shot predictions. Moreover, by having a better predictive model of ball trajectory, people should be able to suppress analyses of much visual information from locations other than the ball, when this information is task irrelevant—although they might continue to tap scene-level movement statistics that contribute to optic flow^22^. Hence, we anticipated a greater amount of alpha-band activity in occipital brain regions in conditions that promote better predictions, as alpha-band activity has been linked to spatial inhibition^17,19,20^.

## Method

### Experiment

#### Participants

Thirty participants were recruited via a first-year research participation scheme at the University of Queensland, Brisbane, Australia. The experiment was restricted to participants with normal vision (as glasses and contact lenses interfered with the eye tracker). Ages ranged from 18 to 29 (*M* = 20.53, *SD* = 2.92). Informed consent was obtained from all subjects prior to testing. This experiment was approved by the University of Queensland ethics committee and conducted in accordance with the Declaration of Helsinki.

#### Stimuli and apparatus

Eighty 5 s basketball videos were encoded from two games of basketball. These all culminated in a jump shot, with the ball reaching the basket on the 137th frame. Video footage had a width subtending ~ 20 degrees of visual angle (dva; 726 pixels), and a height subtending ~ 10 dva (360 pixels), with participants viewing inputs from a distance of 57 cm, directly in front with their chin placed on a rest. Videos were presented on an ASUS VG248QE 3D Monitor, using the Psychophysics Toolbox for Matlab^23^ and custom MatLab 2015b code^24^. The monitor had a resolution of 1920 × 1080 pixels and a refresh rate of 60 Hz, but videos were animated at 30 frames per second. A Cambridge Research Systems LiveTrack fixation monitor was used for eye tracking (sampling monocular eye positions at 30 Hz). A Biosemi International ActiveTwo system was used to record EEG data (sampling rate: 1024 Hz, channel array: 64 AG/agCI, layout: extended international 10–20 system).

#### Procedure

Each participant watched all 80 clips twice, once upright and once inverted. Presentation order was counter-balanced, such that 40 clips were first seen upright, and 40 inverted, so any conditional differences cannot be attributed to practice or learning effects.

At the start of each experimental session the participant performed a nine-point eye tracking calibration task, followed by a short test of the eye tracking calibration (a moving white dot was presented for the participant to follow, with feedback regarding eye gaze presented onscreen), and then a single practice trial. These were used to confirm calibration quality, and explain the task, and were repeated at need.

At the start of each trial participants performed a fixation calibration check. A white fixation dot (0.44 dva) was shown in the centre of the screen, and a green dot (0.48 dva) was used to mark the registered eye gaze position. If the participant was looking at the fixation dot, and the eye tracking dot was on or within 35 pixels (0.97 dva; marked by a blue square) of the fixation dot, they could immediately initiate the trial with a middle mouse button press. If they were fixating and the eye tracking dot was offset, this would indicate to the participant should shift their head position back to the position it had been in during calibration. This insured eye tracking calibration quality was maintained throughout the experiment and allowed for small discrepancies between actual gaze position and recorded gaze position throughout each trial to be corrected.

Directly after the fixation check, the first frame of the video was presented for 1.5 s. During the final second of this period an open, red, shrinking circle (from 2.5 to 0.8 dva) was overlaid, centred on the ball position, to ensure all trials began with accurate gaze toward the ball position. After this, 4.5 s (135 frames) of basketball footage was shown, generally depicting some dribbling and passing, and always culminating in a jump shot. The final frame depicted the ball position 2 frames *before* reaching the basket (or not; i.e. the frame at which you could tell if the ball would go in, or miss). The screen then displayed two options concerning the behavioural judgement, prompting the participant to predict the shot outcome: “Left = Basket”—“Right = Miss”. The participant then received feedback on their response; the last 1 s of the video was shown, which included a replay of 500 ms *prior* to the shot reaching the basket (or not), and 500 ms after. During feedback, the video either had a green (correct) or red (incorrect) frame. A circular arc was then briefly flashed, with an angular subtense indicating the proportion of the experiment that had been completed, with the next video beginning after a ~ 3–4 s delay (see Fig. 1 for a graphic depicting the presentation protocol).

### Data analyses

#### Shot outcome

**Figure 2:**
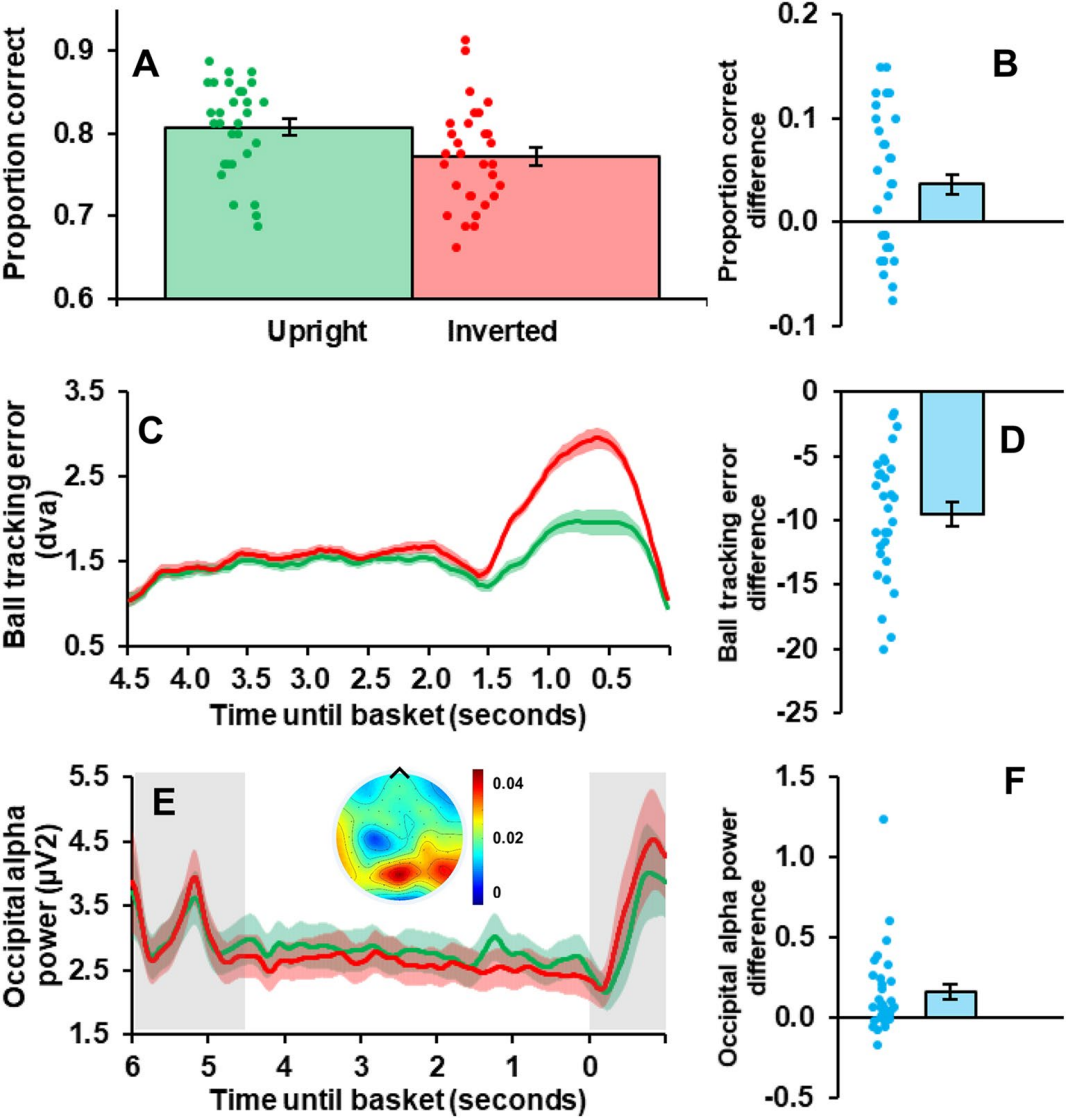
(**A**) Mean proportion correct scores for upright (green) and inverted (red) trials. Coloured dots depict individual participants. Error bars depict ± 1 SE across participants. (**B**) Proportion correct difference scores, taken as upright minus inverted (figure details same as (**A**). (**C**) Mean error (dva), between individual fixation positions and actual ball locations, as a function of time from video onset (seconds) for upright (green) and inverted (red) videos. Shaded regions depict SE across participants. (**D**) Ball tracking error difference scores, taken as upright minus inverted (using the full sample period; figure details same as **A**). (**E**) Time–frequency data showing mean occipital alpha power (µV^2^), as a function of time from shot outcome (0 s) for upright (green) and inverted (red) videos. Coloured shaded regions depict ± 1 SEM across participants. Depicted data include 1.5 secs pre-video onset and 1 s post-video offset (shaded grey regions). Pre-video onset, the ball localising circle was displayed over a static image (the first video frame). Post-video offset the screen was blank and the response screen was displayed. Topographic map depicts conditional difference (upright—inverted) in alpha-band (8–12 Hz) activity for full sample duration. Created using the FieldTrip toolbox for Matlab. **F)** Occipital alpha power difference scores, taken as upright minus inverted (using the full sample period; details as per Fig. 2A). All graphs created using Microsoft Office.

The proportion of trials on which individual participants correctly reported shot outcomes was contrasted for upright and inverted videos, using repeated measures frequentist and Bayesian *t*-tests (*null hypothesis*: no conditional difference, *alternative*: conditional difference present; see Fig. 2A,B). All Bayesian analyses were conducted using JASP, with a default Cauchy prior with width of 0.707, and were interpreted with the Jeffreys’ evidence categories^25^.

#### Ball tracking data

Eye gaze position, taken as screen pixel position relative to screen centre, was recorded for each frame of the videos. The left eye was used primarily, except in cases where calibration proved difficult with the left eye, and so the right eye was used instead. For each trial, eye gaze position data points were corrected relative to the mean fixation position for the last 500 ms before the trial was initiated (during the fixation check step).

For each frame, the error between individual fixation positions and the actual ball location was calculated (as a vector magnitude). For each condition (Upright and Inverted), fixation error was averaged across trials and frames, to provide a single conditional estimate of error. Repeated measures frequentist and Bayesian *t*-tests were used to compare each participant’s mean error scores (in pixels) for upright and inverted trials (*null hypothesis*: no conditional difference, *alternative*: conditional difference present; see Fig. 2C,D).

**Figure 3:**
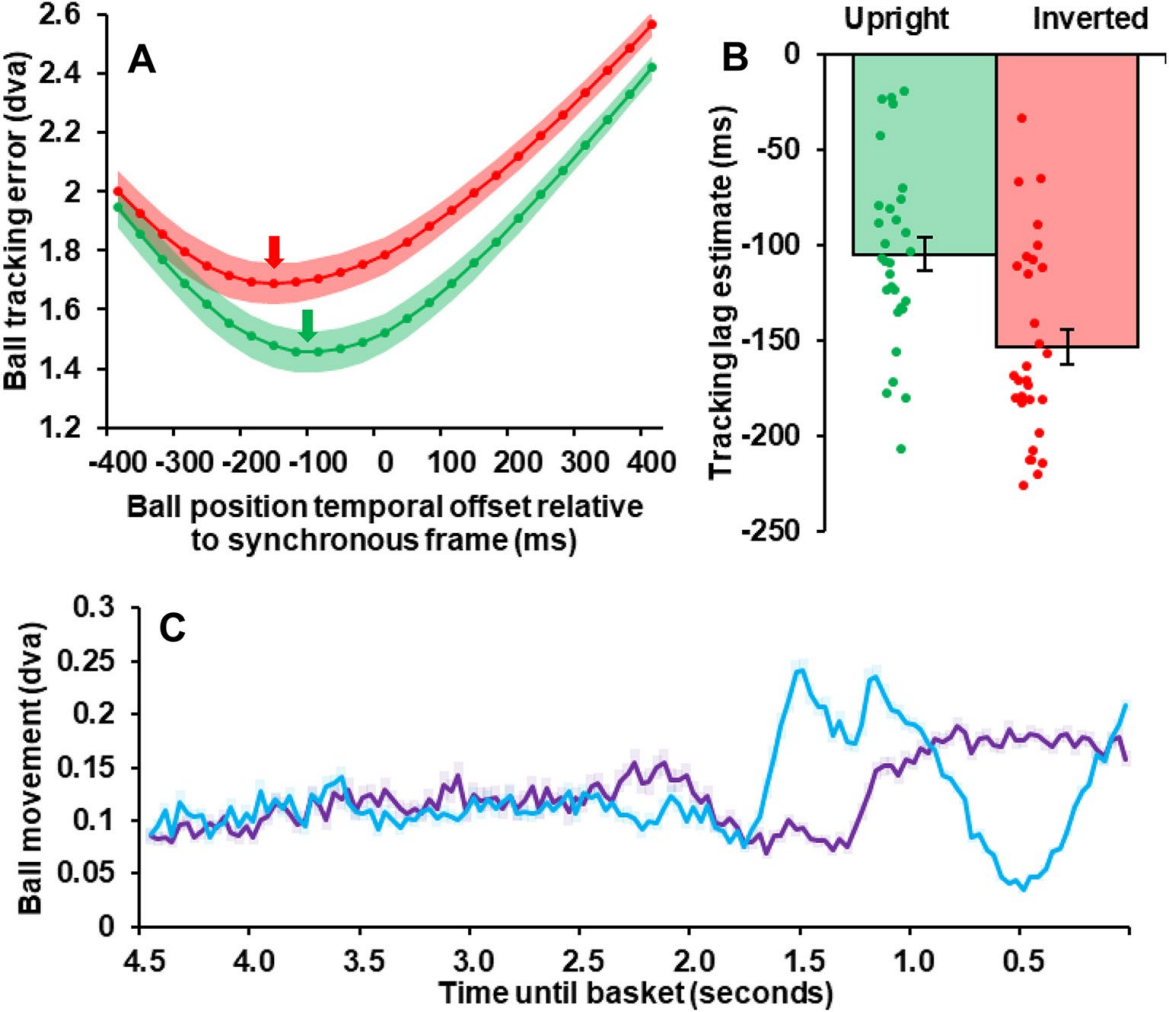
(**A)** Mean tracking error (dva) between gaze and actual ball locations as a function of offset from synchronicity (0 ms) for upright (green) and inverted (red) videos. Negative temporal offsets refer to gaze positions indexed against past positions of the moving ball, whereas positive values are indexed against future positions of the moving ball. Coloured arrows mark ball tracking error minima for each condition. (**B**) Mean tracking lag estimates for upright (green) and inverted (red) trials. Coloured dots depict individual participants. Error bars depict ± 1 SE across participants. (**C**) Mean changes in physical ball positions (dva), as a function of time (seconds) along X- (purple) and Y-axes (blue). Error bars depict ± 1 SEM across videos. Note that videos were presented at 30fps, so y-axis values indicate change per ~ 33 ms intervals.

We repeated tracking error calculations, indexing instantaneous gaze positions against future, present and past ball positions—to estimate how far gaze might *lag* the instantaneous position of the moving ball. For each condition (Upright and Inverted) tracking errors were averaged across trials and video frames, to provide a single estimate of error for each frame offset (from a 400 ms lag, to 400 ms into the future; e.g., a -100 ms offset compares the *current* gaze position to the balls’ position 100 ms earlier; see Fig. 3A).

Polynomial functions (10th degree) were fit to individual data, to find the temporal offset corresponding with *least* error for each participant, separately for each condition. This provided ‘tracking lag estimates’ of how far gaze lagged the instantaneous position of the moving ball (e.g. the past ball position at which gaze seemed to be directed on average, thereby minimizing tracking error). Repeated measures frequentist and Bayesian *t*-tests were used to compare each participant’s tracking lag estimate on upright and inverted trials (*null hypothesis*: no conditional difference, *alternative*: conditional difference present; see Fig. 3B).

#### Frequency data

During pre-processing, data were high-pass (2 Hz), low-pass (100 Hz) and band-stop (45–55 Hz) filtered, using a windowed sinc FIR filter via the FieldTrip toolbox^26^ for Matlab. Data were then subjected to an independent component analysis, implemented by the FieldTrip toolbox for Matlab, to remove blink artefacts. Electrode activity was then average referenced, to correct for baseline skin conductance levels. A multitaper time–frequency transformation based on multiplication in the frequency domain was then conducted on the data from 500 ms before and after the video period on each trial (a 5.5 s period total; taper: hanning; frequencies of interest: 2–14; analysis window centred on all times intervals). Only occipital channel data was saved following this analysis (PO7, PO3, O1, Oz, POz, PO8, PO4, and O2). Trial sorting was then performed.

Repeated measures frequentist and Bayesian *t*-tests were used to compare participant’s mean occipital alpha-band (8–12 Hz) oscillatory activity for upright and inverted trials (*null hypothesis*: no conditional difference, *alternative*: conditional difference present; see Fig. 2E,F). Time–frequency data were averaged across trials, alpha frequencies (8–12 Hz), occipital electrodes (PO7, PO3, O1, Oz, POz, PO8, PO4, and O2), and the trial period (4.5 s of video). An additional two-way repeated measures ANOVA was conducted to establish if occipital alpha-band differences emerged before the onset of video dynamics.

## Results

As expected, we found that overall people were better at predicting jump shot outcomes when footage was upright, as opposed to inverted (paired *t*_29_ = 2.89, *p* = 0.007, *d* = 0.527; with a Bayes factor analysis revealing substantial evidence for the alternative hypothesis—that there would be a conditional difference, *BF*_10_ = 5.88; see Fig. 2A,B). We also found that participants were better at tracking the ball as it moved in upright, as opposed to inverted videos (paired *t*_29_ = 10.59, *p* < 0.001, *d* = 1.934; with a Bayes factor analysis revealing *decisive* evidence for the alternative hypothesis—that there would be a conditional difference, *BF*_10_ > 1000; see Fig. 2C,D). We also found evidence that occipital alpha-band oscillatory activity was *enhanced* while watching upright, as opposed to inverted videos (paired *t*_29_ = 3.22, *p* = 0.003, *d* = 0.588; with a Bayes factor analysis revealing strong evidence for the alternative hypothesis—that there would be a conditional difference, *BF*_10_ = 12.143; see Fig. 2E,F).

As highlighted in Fig. 2E, occipital alpha-band activity was decreased during the dynamic video relative to the 1.5 s sequence when the ball’s position within a static image was highlighted (i.e. the first frame of the forthcoming video; *F*_29_ = 11.508, *p* = 0.002, ƞ_p_^2^ = 0.284). The effect whereby occipital alpha-band activity was greater on upright than inverted trials was qualified by an interaction (*F*_29_ = 5.386, *p* = 0.028, ƞ_p_^2^ = 0.157), wherein this difference was not present during the static image presentation (1.5 s period pre-video onset; *F*_29_ = 0.002, *p* = 0.963)—only during the dynamic video (*F*_29_ = 17.097, *p* < 0.001). Hence our data highlight a disproportionate decrease in occipital oscillatory alpha power on inverted trials while watching video footage.

Analyses of Tracking Lag Estimates revealed that in both conditions instantaneous gaze *lagged* the true position of the moving ball, but this lag was smaller for upright (M ~ 104 ms, SD ~ 48 ms) than for inverted (M ~ 153 ms, SD ~ 51 ms) videos (paired *t*_29_ = 4.24, *p* < 0.001, *d* = 0.774; see Fig. 3A). A Bayes factor analysis of these data revealed *decisive* evidence for the alternative hypothesis—that there would be a conditional difference (*BF*_10_ = 135; see Fig. 3B). Figure 3C depicts frame-by-frame ball position change throughout the average video, along the X- (purple) and Y-axis (blue). This highlights a vertical acceleration at the beginning of the shot arc, a deceleration at the arc peak, and an acceleration towards the hoop.

**Figure 4:**
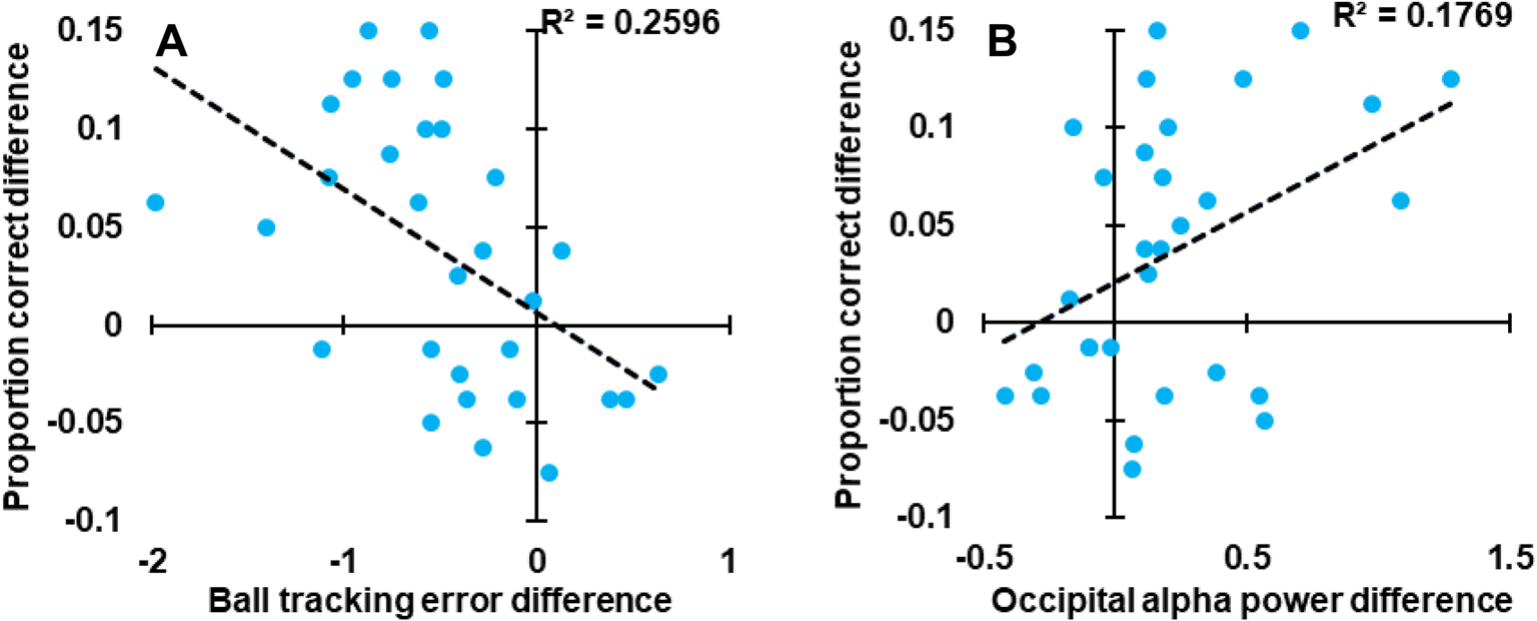
Conditional differences (upright minus inverted) in proportion correct as a function of differences in **(A)** ball tracking error and **(B)** occipital alpha power, in the last 500 ms of each video, for each participant.

Conditional differences (upright minus inverted) in participants’ ability to track the ball in the 500 ms period leading up to a jump shot outcome were *negatively* associated with conditional differences in peoples’ ability to predict shot outomes (*R*^*2*^ = 0.260, *p* = 0.004; with a Bayes factor analysis revealing strong evidence for the alternative hypothesis—that there would be a linear relationship between the two sets of conditional differences, *BF*_10_ = 11.723; see Fig. 4A). In contrast, conditional differences in occipital alpha activity in the 500 ms period leading up to a shot outcome were *positively* associtated with conditional differences in peoples’ ability to predict shot outomes (*R*^*2*^ = 0.177, *p* = 0.021; with a Bayes factor analysis revealing anecdotal evidence for the alternative hypothesis—that there would be a linear relationship between the two sets of conditional differences, *BF*_10_ = 2.926; see Fig. 4B). We checked to see if these relationships were present for an equal length period at video onset, and found no correlation between ball tracking error difference or occipital alpha power differences and predictive accuracy differences (*R*^*2*^ = 0.288, *p* = 0.123, & *R*^*2*^ = 0.102, *p* = 0.590; with Bayes factor analyses revealing anecdotal & substantial evidence for the null hypothesis—that there would be no linear relationship between each set of conditional differences, *BF*_10_ = 0.707, & *BF*_10_ = 0.261).

The conditional differences we have identified are potentially confounded by differences in gaze position—particularly along the y-image axis. This consideration is problematic when considering our findings regarding occipital alpha-power, as this has been linked to changes in gaze and attentional directions^27,28^. We found, however, that conditional differences in physical Y-axis gaze position were not associated with differences in alpha power (*R*^*2*^ = 0.272, *p* = 0.145; with a Bayes factor analysis revealing anecdotal evidence for the null hypothesis—that there would be no linear relationship between the two sets of conditional differences, *BF*_10_ = 0.624). Nor did we find an association between Y-axis gaze positions and ball tracking error (*R*^*2*^ = 0.166, *p* = 0.381; with a Bayes factor analysis revealing substantial evidence for the null hypothesis—that there would be no linear relationship between these measures, *BF*_10_ = 0.327). Nor was there a relationship between Y-axis gaze positions and predictive performance (*R*^*2*^ = 0.138, *p* = 0.467; with a Bayes factor analysis revealing substantial evidence for the null hypothesis—that there would be no linear relationship between these datasets, *BF*_10_ = 0.292). Finally, a hierarchical multiple regression revealed that occipital alpha power differences still predicted predictive performance after controlling for the effect of Y-axis gave position differences (β = 0.412, *p* = 0.031). These analyses were conducted using the last 500 ms of trial data, when physical gaze position differences were maximised, and where we find evidence for a relationship between predictive accuracy, ball tracking error, and alpha power.

## Discussion

In line with our hypotheses, we found that for upright videos people were better at predicting shot outcomes and tracking the ball as it moved, and they had enhanced occipital alpha-band oscillatory activity. Advantages for predicting upright shot outcomes were associated with increased ball tracking accuracy and increases in alpha-band activity in occipital brain regions.

We believe participants were better able to predict shot outcomes, and track moving balls, in upright than in inverted videos because upright presentations accord with peoples’ expectations^1–4^. Our work would therefore build on previous findings suggesting the control of eye movements, while viewing or participating in sport, relies on the formation of an accurate predictive model of the visual environment^4,6,9^. We this predictive model is disrupted via scene inversion, peoples’ ability to predict jump shot outcomes was impaired (see Fig. 2A,B), and people were less able to track moving balls (see Fig. 2C,D), resulting in an increased lag of gaze relative to the instantaneous position of the moving ball (see Fig. 3A,B).

Our paradigm builds on past investigations, by adding time–frequency analyses of EEG recordings. We found that occipital alpha-band activity generally decreased following the onset of video dynamics, and this decrease was greater for inverted footage. We also found that people were better able to predict shot outcomes on upright trials, and this was associated with *enhanced* alpha-band activity in occipital brain regions (see Fig. 2E,F). We had anticipated this relationship, as enhanced alpha-band activity has been linked to selective visual attention^13–17^ and spatially-mapped inhibition of visual information processing^17,19,20^; although, see^21^. We had reasoned that by having a better predictive model of the dynamics of the environment, people would be better able to suppress task irrelevant information from all positions *other* than the ball and its direct surrounds. Hence, we had anticipated enhanced alpha-band activity in conditions that promote better predictions. It is, however, also conceivable that enhanced occipital alpha-band activity and better predictions could have ensued from an upregulation of task-relevant information^21^. In either case, having an accurate internal predictive model of the world would help the observer parse task-relevant from task-irrelevant information, allowing the brain to prioritise processing of the former.

Alpha power modulations were evidently associated with gaze direction in our experiment—raising the question as to whether conditional differences in alpha power could have been related to input simple image statistics (e.g.^27,28^), as opposed to some internal cognitive process(es) that are involved in predicting dynamics. However, our data also reveal a robust relationship between individual differences in occipital alpha-band activity (for upright and inverted videos) and individual differences in predictive performance (see Fig. 4B)—so it would seem that alpha-band differences were indeed related to a cognitive process involved in predicting jump shot outcomes. We also found that while ball tracking error and alpha power could predict predictive performance, none of these variables could be predicted by Y-axis gaze positions, and alpha power still predicted performance after controlling for the effect of Y-axis gaze position. Moreover, all these analyses targeted the final 500 ms of trial data, when ball movement was greatest along the Y-image axis (so the impact of gravity on ball trajectory would be greatest), and when people had to perform our predictive task. Alpha differences from earlier epochs did not predict jump shot predictions. The fact that we see a temporally reasonable relationship between alpha power differences and predictive behaviour strengthens our view that these measures are contingently related.

We note that there are little conditional differences in ball tracking error and alpha power at early stages of video presentations. We believe this is because ball movements, particularly along the Y-image axis, were much reduced at these times, relative to the final stages of video presentations (see Fig. 3C). There may therefore be less need to suppress information from extraneous positions in these circumstances, as both the amount of positional change is reduced, and because the key predictive task has not yet been attempted. Key correlates of prediction accuracy could be heightened at the final stages of each presentation, and largely absent earlier, as the better predictive model of environmental dynamics available to upright presentations becomes pertinent, both due to task demands and increased Y-axis ball movement. The improved predictive model, and its consequences for inhibitory processes at later stages of video presentations, need not have a linear consequence in terms of neural activity (for a non-linear increase in alpha power as a function of predictability, see^29^).

The coarse spatial resolution of EEG analyses are consistent with our having detected a process that is widely dispersed across occipital cortex, such as the suppression of visual information from the majority of the scene—other than the predicted locations of the moving ball. We would not anticipate a linear relationship between the amount of suppression and increases in ball tracking error. A small increase in tracking error could be associated with a widely dispersed reduction in suppression—as many more potential future ball positions would need to be monitored given even a slight increase in uncertainty regarding ball trajectory.

There are several factors that might have contributed to poorer predictive performance and ball tracking on inverted trials. We suggest one factor was the reversal of the expected impact of gravity during inverted videos^10–12^. In favour of this, we note that ball tracking was especially disrupted *late* during inverted trials (see Fig. 2C), as the ball arced vertically through the air toward the hoop (see Fig. 3C). Moreover, both ball tracking error and conditional alpha differences during this late period were associated with predictive performance differences (see Fig. 4), whereas these factors were not so associated at earlier epochs of stimulus presentations (when the ball was not differentially accelerating along the y-axis; see Fig. 3C). So, we believe that expectations about gravity have played a substantive role in our data.

Changes to the spatial distribution of people and objects in inverted videos might also have contributed to our patterns of results. Neural representations are sharper, and people are more sensitive to objects when they are presented in retinal locations that correspond to where they would typically appear in the world (e.g., planes in the upper visual field; for review of ‘scene-location’ congruency, see^30^). In inverted videos, the positions of players relative to the hoop, to each other, and to the crowd would all be foreign. Thus, there may have been more neural and perceptual sharpening for objects in upright relative to inverted video presentations. We do not see these as a conflicting set of interpretations relative to our emphasis on the effects of gravity, but rather see these as inter-related consequences of disrupting people’s internal models of the world.

We have suggested that the advantage afforded to inputs consistent with internal predictive models is caused by the brain being better able to suppress task irrelevant information (from positions other than the immediate predicted location of the ball as it moves). This is similar to neural ‘sharpening’ models and to data that suggests enhanced sensory processing for inputs that conform to expectations, and suppression for inputs that do not conform to expectations^31–33^.

Similar ideas to our suggestion of a suppression of task irrelevant information have been advanced in literature concerning how expertise modulates cortical activity—an area with clear parallels to our task (i.e., people are novices at viewing the world upside-down, and expert at viewing upright images and footage). Experts, relative to novices, show *enhanced* alpha power on tasks such as map reading^33^ and rifle shooting^34^. Experts also show more economical cortical activations^35^ and can better inhibit attentional shifts toward task-irrelevant inputs during top-down, goal-directed behaviours^36^. A commonality to all these observations is that when people are well attuned to a visual task, they can better suppress analyses of irrelevant visual information, an act that tends to be associated with *enhanced* alpha power in occipital brain regions. One aspect of our data suggests modulations of information processing can be anticipatory.

We have shown, using naturalistic stimuli in a novel behavioural paradigm, that peoples’ ability to predict the near future can be impaired via inversion. We link this to a disruption of the brain’s predictive model of the world’s dynamics, particularly concerning expectations about the impact of gravity^10–12^ and the spatial distribution of objects^28^. This had a similar deleterious impact on ball tracking, which has previously been linked to predictive models in the visual brain^4,6,9^. Finally, we found that the power of changes in occipital alpha-band activity tend to scale with changes in predictive accuracy. Overall, our data are consistent with a relationship wherein the formation of a successful predictive model of the environment allows people to better select relevant information for processing, and to suppress analyses of other visual information as irrelevant to the task at hand. This implies that when sportspeople are ‘in the zone’ and performing well, they might be more successful in suppressing analyses of task irrelevant visual information—in a situation where *less* processing of visual information is more.
